# Case study of a method of development of a selection process for community health workers in sub-Saharan Africa

**DOI:** 10.1186/s12960-019-0412-2

**Published:** 2019-10-25

**Authors:** Celia Brown, Richard Lilford, Frances Griffiths, Prince Oppong-Darko, Myness Ndambo, Marion Okoh-Owusu, Emily Wroe

**Affiliations:** 1 Division of Health Sciences, Warwick Medical School, Coventry, United Kingdom; 2 Ghana Health Service, Ellembelle, Ghana; 3 Partners In Health, Neno, Malawi; 4 Division of Global Health Equity, Brigham & Women’s Hospital, Boston, United States of America

**Keywords:** Community health workers, Selection, Recruitment, Performance

## Abstract

**Background:**

Choosing *who* should be recruited as a community health worker (CHW) is an important task, for their future performance partly depends on their ability to learn the required knowledge and skills, and their personal attributes. Developing a fair and effective selection process for CHWs is a challenging task, and reports of attempts to do so are rare. This paper describes a five-stage process of development and initial testing of a CHW selection process in two CHW programmes, one in Malawi and one in Ghana, highlighting the lessons learned at each stage and offering recommendations to other CHW programme providers seeking to develop their own selection processes.

**Case presentation:**

The five stages of selection process development were as follows: (1) review an existing selection process, (2) conduct a job analysis, (3) elicit stakeholder opinions, (4) co-design the selection process and (5) test the selection process. Good practice in selection process development from the human resource literature and the principles of co-design were considered throughout. Validity, reliability, fairness, acceptability and feasibility—the determinants of selection process utility—were considered as appropriate during stages 1 to 4 and used to guide the testing in stage 5. The selection methods used by each local team were a written test and a short interview.

**Conclusions:**

Working with stakeholders, including CHWs, helped to ensure the acceptability of the selection processes developed. Expectations of intensiveness—in particular the number of interviewers—needed to be managed as resources for selection are limited, and CHWs reported that any form of interview may be stressful. Testing highlighted the importance of piloting with CHWs to ensure clarity of wording of questions, interviewer training to maximise inter-rater reliability and the provision of guidance to applicants in advance of any selection events. Trade-offs between the different components of selection process utility are also likely to be required. Further refinements and evaluation of predictive validity (i.e. a sixth stage of development) would be recommended before roll-out.

## Background

Community health workers (CHWs) are a vital cadre within the health system [1–3], linking communities with health systems. CHWs can contribute effectively to healthcare provision [4], but they are not a panacea to the lack of human resources for health, and particularly not to the lack of highly skilled healthcare workers [5, 6]. This paper focuses on CHWs in low- and middle-income countries (LMICs), where CHWs’ work has previously focused on single health conditions such as HIV or vaccination. However, their roles are now widening to incorporate surveillance, referral, education/support and treatment for multiple health conditions [7, 8]. CHW programmes are also expanding in size, increasing the number of CHWs and the proportion of the population with access to a CHW as part of the drive towards universal health coverage [9].

Being a CHW is often seen as a good job opportunity [10], so there is usually no shortage of applicants for CHW posts [11]. As with any cadre of healthcare staff, it is essential to recruit the most appropriate CHWs for the role. This may not always occur in practice, as there is evidence that some CHWs struggle to cope with the workload [12], leading to poor retention [13, 14] and sub-optimal effectiveness [5, 6], although these challenges may also be due to other factors including unrealistic expectations of CHWs and poor supervision [6, 15]. The most appropriate CHWs are those who possess the necessary knowledge, skills and attributes (KSAs) required. These KSAs may include time management, respect, kindness, empathy, diligence and acceptance by the community [5, 16]. The specific KSAs required for a particular CHW programme and their relative importance will depend, in part, on the exact design of the programme and CHWs’ expected roles and tasks. In terms of relative importance, some KSAs may be considered “essential”, while others are “important” or “supplementary”. Furthermore, some of these KSAs can be developed through CHW training, but the time available for training is often short [17], and some KSAs are less amenable to training than others. As a result, it could be hypothesised CHWs who can demonstrate, at selection, the essential KSAs and/or those that are relatively difficult to teach during the short CHW training period are likely to perform better on-the-job and help maximise the contribution of a CHW programme to health outcomes.

Traditionally, CHWs were selected by their own communities. However, there is evidence that selection that relies entirely on community decision-making is not optimal: it may be unfair (subject to nepotism) [18–20] or ineffective (i.e. it may not select for essential KSAs and those that are more challenging to teach using a valid and reliable method) [18, 21]. Thus, as CHW programmes increasingly come within the remit of government health ministries, selection processes that include, but are not restricted to, community representation are being developed. Within this changing context, formal selection processes are becoming an important component of CHW programmes. Two recent reports—a WHO guideline on optimising CHW programmes [22] and the CHW *Assessment and Improvement Matrix* (CHW AIM) [23]—both include appropriate and effective CHW selection as a contributor to a highly functioning CHW programme. The WHO recommends using selection criteria based on education and personal attributes, and notes the importance of community engagement. However, the certainty of the evidence base used as part of the development of the WHO’s recommendations was weak, and no specific selection methods were considered [22]. CHW AIM, meanwhile, states that a “highly functional” CHW programme involves communities in recruitment and ensures that the “attitudes, expertise and availability [KSAs] deemed essential for the job are clearly delineated prior to recruitment and linked to specific interview questions/competency demonstrations” [23]. This delineation of the expected or required KSAs as a job specification is therefore an important pre-requisite to designing an effective selection process. Although CHW AIM provide examples of selection methods, there is no strong evidence to favour one method (or combination of methods) over another [11, 19, 24], and the evidence base is limited by poor descriptions of selection processes [15, 25].

How can the CHW AIM recommendation be implemented in practice? In this paper, we report a case study of the development of a selection process for CHWs in two settings in sub-Saharan Africa, in which we sought to identify which KSAs should be demonstrated at selection and subsequently translate these into specific questions within a selection process. Our approach was based on three guiding principles:

1. An appreciation of good practice in selection process development, as found in the human resource literature, such as the work of Evers et al. [26].
2. The benefits of an experienced-based co-design approach [27], with iterative cycles of development, testing and review.
3. The need to consider the concepts of validity, reliability, fairness, acceptability and feasibility [28].

We were fortunate enough to obtain external funding from the UK Medical Research Council to undertake this work. Most CHW programmes will not have access to similar levels of funding for selection process development. Nevertheless, it is important to disseminate our work for three reasons. The first is that other CHW programme providers may wish to use a subset of the methods reported here, and we have therefore provided our data collection templates as supplementary materials. The second is that the components of the selection processes developed such as test or interview questions (also provided as supplementary materials) have been pre-tested and could be adapted for use by others without significant further pre-testing. The third is to share the lessons learned from our work and to suggest a low-resource approach to selection process development for others to use based on our learning. This approach is not “free”, but we believe that investing in selection will pay off, for it provides the foundations for the human capital needed for a successful CHW programme [29].

## Case presentation

### Settings

The work described here is a collaboration between academic institutions and CHW programmes. The two CHW programmes are expanding in terms of the number of CHWs employed, making recruitment and selection key tasks. Both programmes were seeking to review and potentially improve their current approach to selection, to help ensure that new recruits would provide a high standard of care. The two programmes operate in different contexts and have different selection processes currently in place, as summarised in Table 1.

**Table 1:** Summaries of CHW programmes for which selection processes were developed

	Neno, Malawi (Partners In Health, PIH)	Ellembelle, Ghana (Ghana Health Service)
Location	Rural – south-west of Malawi on the border with Mozambique	Rural – Western Region of Ghana
CHW programme implementors	PIH, a US-based non-governmental organisation, working with the Ministry of Health in Neno	Youth Employment Agency and District Health Directorate according to the Ghanaian CHW Roadmap document [30]
Number of CHWs	Currently being increased from 1 050 to 1 200 by 2019	Currently being increased from 95 to a target of 1 000 by 2026
Payments to CHWs	Monthly stipend	Monthly salary
CHW tasks	Integrated “household model” [31] Timely case-finding Linkage to care On-going support and accompaniment of patients in care and tracking missed visits Health education	Health education—malaria Detection and management—malnutrition Home visits—family planning First aid for household emergencies Community education—injury prevention Detection of danger signs in children and early referral
Average education level of CHWs	5–6 years of primary education	High school leavers
Current selection process	Community meeting Community nomination Literacy test	Community recruitment and local ownership Call for submission of applications Nomination by a chief, queen mother, Member of Parliament or other prominent community member Interview by community leaders
Selection criteria	Resides in village or community, known and trusted community member with approval from community, literacy in Chichewa and able to read and complete data forms and health messaging, able to commit 20 h per week, good communication skills for relaying health education, able to travel long distances on foot, ability to relate to and support community members.	Resides in community or lives close by, speaks English and the local language fluently, free from any criminal and behavioural records, endorsed by the community for responsible and respectable behaviour and educated to at least Junior High School level (including ability to read and write).

### Approach to selection process development

We used a five-stage approach to developing the selection process: (1) review an existing selection process, (2) conduct a job analysis, (3) elicit stakeholder opinions, (4) co-design the selection process (including “alpha”, or basic “could this work?” testing), and (5) test selection process (“beta”, or pre-pilot testing). An early decision was made to focus on a written test and a short face-to-face interview as the selection methods, rather than alternatives such as a multiple mini-interview, which would have been challenging to administer in a CHW programme setting.

#### Stage 1: Review an existing selection process

There is no point in designing a new selection process from scratch if an appropriate process already exists that could be easily adapted for a new setting. We therefore examined the predictive validity of an existing selection process used by *Living Goods* for their CHW programme in Kenya in order to identify elements that may work well in our settings; the decision to use this process was opportunistic given the contacts of the research team. The *Living Goods* CHW programme combines door-to-door health care with sales of products such as fortified flour and solar lights. The *Living Goods* selection process includes a written test of business maths, written comprehension and personal attributes, and a face-to-face interview which assesses motivation to be a CHW and ability to sell. The results of the analysis of predictive validity are described elsewhere [32]; in summary, no element achieved a correlation coefficient of 0.3 considered to be the minimum for an adequate predictor of on-the-job performance [33], although the written elements were more predictive than the interview elements, and these were taken forward to stage 4 as described below. The lessons learned during this and subsequent stages of the project are shown in Table 2.

**Table 2:** Lessons learned during selection process development

Stage	Process	Lesson
1	Review existing selection process	An existing process (or processes) provide(s) ideas and examples but may not necessarily be effective even within the setting for which they were designed: evaluating predictive validity is important.
2	Observational job analysis	Use each household visit/client contact/CHW activity as the “unit” if such activities are all of a similar duration, or a suitable time block (e.g. 10–15 min) if the activities undertaken vary in duration. Include data collection on the type of activity as this may help the choice of scenario topics for questions developed.
3	Obtaining views of stakeholders	Include CHWs if pre-implementation testing is limited—we obtained a considerable amount of feedback from CHWs during the process of training field workers to undertake the cognitive interviews.
3	Data analysis of sorting task	A different approach (to that planned and therefore used here) may be warranted given that no KSAs were identified for inclusion based on the data from Ghana. For example, excellent and important ratings could be combined, or stakeholders could be asked to select the five most important KSAs.
3	Managing stakeholders’ expectations	Stakeholders may have unrealistic expectations of the resources available for selection in terms of how many people should sit on an interview panel (a panel with multiple interviewers may also be unacceptable to CHWs, with some reporting in alpha and beta testing that any kind of interview would be very stressful).
4	Writing high quality questions	Allow sufficient time and be prepared for the process to be a little challenging.
4	Determining and operationalising marking/scoring criteria for interview questions	Criteria need to be explicit and interviewer training is essential to ensure fairness across CHWs interviewed by different individuals.
5	Determining test length	The length of test that is feasible for CHW programmes may not be sufficiently reliable and thus a trade-off between reliability and feasibility may be required.
5	Deciding how to combine scores from different elements of a selection process	Given the low correlations between scores on each element, a low passing score for the written test may be appropriate, followed by combining scores for final selection decisions. However, this may increase the number of applicants shortlisted for interview to an unmanageable number.
5	Ensuring content validity	Keep questions as simple as possible and double-check that they relate to CHWs’ on-the-job roles (with CHWs if possible).
5	Enhancing fairness and applicant acceptability	Provide applicants with information about the selection process in advance, possibly including some example questions.

#### Stage 2: Conduct a job analysis

**Fig. 1:**
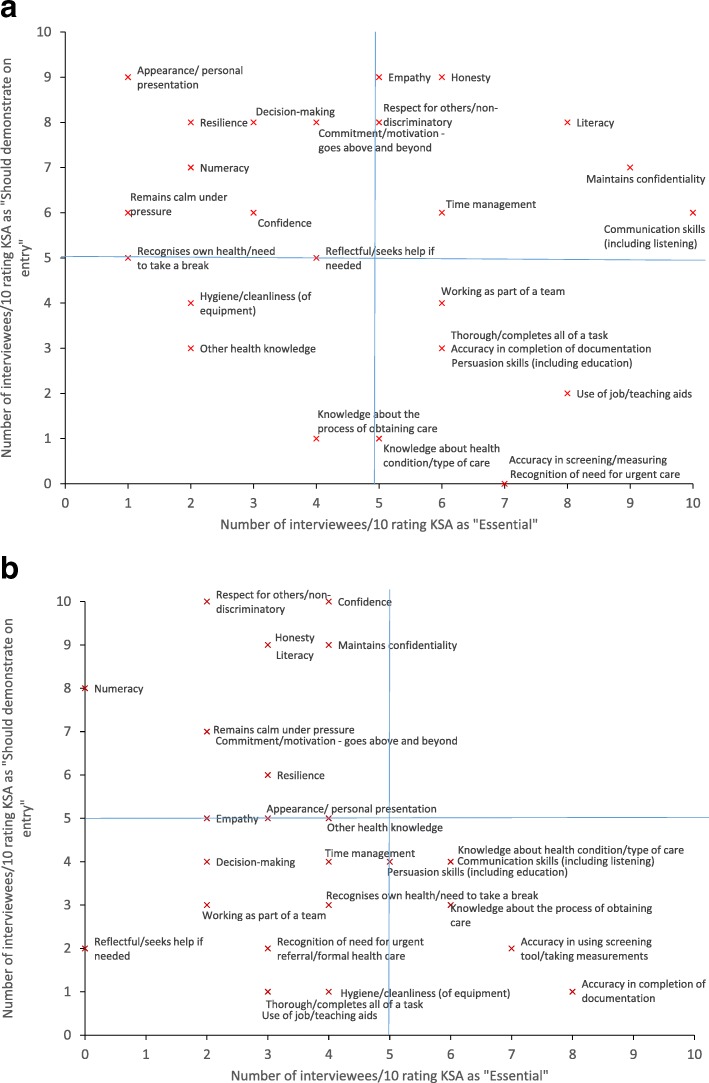
**a** Stakeholder sorting task results: Malawi. **b** Stakeholder sorting task results: Ghana

We used an observational approach to job analysis [34]. A purposeful sample of six CHWs was identified in Malawi and ten in Ghana, who were subjectively identified by their supervisors and managers as being high performers. Purposeful sampling enabled us to elicit information on the KSAs demonstrated by those considered excellent in their work and thus those that should be considered for inclusion as selection criteria. The CHWs identified were contacted and asked if they would volunteer to participate in the study. Each CHW was observed for 2 days. The project field worker in each setting was trained on observational job analysis for 2 days before commencing data collection, which used the structured proforma shown in Additional file 2. The field workers then shadowed each CHW, noting the specific KSAs demonstrated, either at any point during each day (Malawi) or during each specific activity lasting at least 10 min (Ghana). The list of the 27 potential KSAs (shown on the *x*-axis on Fig. 1) was developed from those considered important for health care staff in general [35–37], supplemented by discussions with the CHW programme teams during project set-up. A long list of potential KSAs was used to help avoid missing any.

Sixteen of the 27 KSAs were demonstrated by all six CHWs in Malawi on both days of observation, suggesting that CHWs used a broad range of KSAs at least once every day. However, the low variability made it difficult to identify which KSAs should be included in the selection process. The KSAs demonstrated in at least half of the KSA logs for Ghanaian CHWs—making them strong candidates for inclusion as selection criteria—were as follows: knowledge about condition/type of care, knowledge about care process, communication skills, persuasion skills (including education) and confidence. Both field workers also noted that CHWs spent the largest proportion of their working day (around 50%) providing household counselling/education including health promotion, and the Malawi team decided to base their question scenarios on this activity.

#### Stage 3: Elicit opinions of key stakeholders

Ten structured interviews with key stakeholders were undertaken by the field worker in each setting (interview guide in Additional file 2). The stakeholders consulted included CHW supervisors and community health nurses, health care professionals who treat those referred by CHWs, CHW programme staff, community assembly members/counsellors and a representative from the Youth Employment Agency (Ghana only). CHWs were not included as stakeholders at this stage as their views were sought during alpha and beta testing.

Each interviewee was asked to undertake a sorting task based on the job-element method [34], in which they rated each KSA used in the job analysis described in stage 2 in two ways: (1) as “could be trained in” or “should be demonstrated on entry to training”; and (2) as “essential”, “important” or “nice-to-have”. The results are shown in Fig. 1 a (Malawi) and b (Ghana). Each diagram shows the number of interviewees (out of 10) rating each KSA as “essential” on the *x*-axis and as “should demonstrate on entry to training” on the *y*-axis. The most important KSAs to include as selection criteria are those in the top right quadrant of the diagram (with at least five interviewees rating the KSA as both “essential” and “should demonstrate on entry to training”). For Malawi, these KSAs were empathy, honesty, respect for others/non-discriminatory, literacy, time management, maintains confidentiality and communication skills. No KSAs were located in this area for Ghana.

The interviews also sought to elicit opinions regarding operationalisation of selection including the length of the test and interview and the composition of the interview panel. Interviews were transcribed; following which, opinions on these operationalisation decisions were identified by CB. Stakeholders thought that the time needed for the existing *Living Goods* written test (total 12 questions) should be around 1 h in Malawi and 30 min in Ghana; with the difference primarily due to differences in previous educational levels across settings. There was a smaller difference when considering the *Living Goods* interview (total eight questions), with a time needed of around 35 min in Malawi and 20 min in Ghana. Stakeholders in Malawi wanted an interview panel comprising two to four individuals and those in Ghana three to five individuals. Stakeholders in both settings envisaged the written test would be conducted using pen/pencil and paper.

#### Stage 4: Co-design of selection process

A 2-day workshop in Nairobi was attended by representatives from all three CHW programmes involved in the study. These representatives included CHW programme managers and other local staff involved in running the CHW programmes, as well as the project field workers. The similarities and differences between the organisation and implementation of the three programmes were identified, and the results of stages 1–3 presented and discussed. The teams from the two settings for which the selection processes were being developed then worked to develop their own blueprints. Each blueprint identified which KSAs would be assessed during the written test and which during the interview. The next step was to produce draft versions of the written test and interview, with specific questions that addressed the KSAs selected. To do so, the teams drew on the existing *Living Goods* tools, evidence from stages 1–3 and their own approaches to selection and experiences of working with CHWs. The teams swapped their drafts and provided feedback, including identifying which KSA was being assessed in each question, for specific feedback on content validity (whether the intended blueprint could be independently matched to the questions).

The teams then discussed how the selection processes would be operationalised in practice. These discussions centred on feasibility in terms of time and the number of people required to be present as interviewers. Local teams thought that using the stakeholders’ recommendations would put too much strain on resource-constrained CHW programmes, so relatively short interviews with one or two interviewers were proposed. However, the pencil and paper format for the written test (without calculators or mobile phones, which may not be available to CHWs in the field all of the time, and to ensure that CHWs would be able to check that the answer given on a calculator was correct) was agreed by both teams.

Following this first workshop, each team used the feedback to refine their selection processes independently and sent it for further feedback to CB and to others involved in their local CHW programmes but who had not been involved thus far. A 1-day local workshop was then held in each setting, during which this further feedback was provided and refinements made. Preliminary decisions regarding marking, scoring and any minimum (passing) scores were made collaboratively, but all such decisions were planned to be reviewed following alpha testing (described below). During the local workshops, the field workers were also trained in cognitive interviewing (to be used in beta testing), and the practice interviews conducted during this training provided feedback from CHWs and other local individuals with similar levels of education. This led to further refinements of the selection process prior to alpha testing.

A small-scale alpha test was undertaken with ten CHWs in each setting, who volunteered to participate. The aim was to invite those new to the role to participate, to provide the best match to those applying for the role. Unlike the CHW observations, the aim was to achieve a random sample across the CHW performance spectrum, with balanced numbers of males/females and previous education levels. No time limit on the written test was imposed, but CHWs were timed, and the length of each interview was recorded. The tests were marked by the field worker and the marks checked by another CHW team member before being entered into a Microsoft Excel database. Two interviewers independently rated each CHW on each interview question and the scores of each interviewer were also entered into the Excel database. CHWs were asked to complete a brief paper questionnaire after the written test and again after the interview to ask for their opinion on each. These questionnaires were anonymous.

Analysis of the mean scores for individual questions suggested the need to review some of the written test questions, either where all CHWs answered correctly or where the mean score was very low. Inter-rater reliability for the interviews was poor, with low kappa coefficients (median across four questions in Malawi 0.15 and across eight questions in Ghana 0.50). The questionnaire-based feedback from CHWs was generally very positive.

CB provided feedback on the results of alpha testing to each local team and further changes to the questions were made if required. The marking criteria for the interview questions were expanded to help improve inter-rater reliability, and more extensive interviewer training was planned prior to beta testing. A final blueprint was produced for the written test and interview in each setting (Additional file 1) and operationalization decisions for beta testing made (Table 3).

**Table 3:** Selection process operationalisation decisions for beta testing

	Malawi		Ghana	
	Written test	Interview	Written test	Interview
Time allowed/expected	45 min	10 min	30 min	5 min
Number of interviewers	N/A	3*	N/A	2*
Minimum (passing) score**	8/12 AND 2/4 in each section	7/10	Not determined	Not determined

*For the purposes of selection process development only (only one interviewer would be present if the interview was used in practice)

**These minimum scores were not applied during beta testing

#### Stage 5: Beta testing of selection process

During beta testing, 20 CHWs in each setting participated in a mock selection process. As in stage 4, the aim was to invite those new to the role to participate, balancing on-the-job performance, gender and previous education where possible, and all participants were volunteers. A time limit on the written test was imposed based on the results of the alpha testing, but all participants also completed the interview (not just those passing the written test). CHWs were again asked to complete a brief, anonymous questionnaire about the test and interview. The data were processed and analysed for further feedback to the local teams.

The internal consistency of the written tests and interviews, as measured by Cronbach’s alpha, ranged from 0.53 to 0.69, suggesting *sub-optimal reliability* (ideally alpha values would be in the range of 0.7–0.9 [38]). The time allowed for the written test appeared to be more than adequate in Malawi and just about sufficient to enable CHWs to complete the test in Ghana. There was a low correlation between written and interview scores (Kendall’s tau-b 0.20 (*p* = 0.28) in Malawi and (− 0.02 (*p* = 0.95) in Ghana, suggesting different KSAs were being assessed in each. There was better inter-rater reliability for the interview questions compared to during alpha testing (median Krippendorff’s alpha [39] across four questions of 0.49 in Malawi; only one score differed across all questions and CHWs in Ghana), suggesting that the additional marking guidelines and interviewer training had been effective.

CHWs generally reported that the test and interview included relevant questions, were fairly easy and were fair. One CHW from Ghana reported that they would not have applied had the written test been used, and 17/20 (85%) thought the interview was too short. One/three CHWs from Malawi reported that they would not have applied had the written test/interview been used. The main concern was that the interview was very stressful and, as a result, may not be fair.

A small “think aloud” cognitive interview was conducted with five further CHWs in each setting, who were asked to explain their reasoning as they answered each question (interview schedule in Additional file 2). Interviews were translated and transcribed for analysis in order to identify any questions where the meaning derived by CHWs was different to that intended by the question writers and which therefore required further review. The interviews revealed that CHWs reported that they used their own (sometimes erroneous) knowledge and job experiences to answer the questions, although this may not occur if the test is used in practice, as respondents would not have any experience as a CHW. A need for clarity on the situational judgement type of items was identified in both settings, so that applicants know whether to respond with what they *would* do (behavioural tendency) or what they *should* do (knowledge). The question asking applicants to use percentages on the Ghana written test was considered difficult; respondents reported that they only use basic addition and subtraction in their work, hence questioning the *validity* of the question. Finally, the question including a double negative on the Malawi test caused particular confusion and may need to be reviewed, as it appeared to be assessing literacy rather than conscientiousness.

Combined with the quantitative analysis of the individual questions, information from the cognitive interviews therefore enabled identification of the sections/questions on the written tests that require further review by local teams before further use, as summarised in Table 4. There was no evidence that any existing interview questions required further review.

**Table 4:** Written test sections/questions requiring additional review (the full question text can be found in Additional file 1)

Country	Section/question	Rationale for review	Suggestions
Both	Comprehension	CHWs using own knowledge/experience to answer questions	Keep as is; the instructions are to use the passage to answer the questions Use a scenario about a topic no respondents would have knowledge or experience of (although this may make the scenario seem less relevant)
Both	Situational judgement	Respondents need clarity as to whether the question is asking *would* or *should*	Use either *would* or *should* for all questions in this section
Malawi	Q2	Poor discrimination—may be too complicated for CHWs	Make slightly easier
Malawi	Q8	Low facility and confusing as double negative	Revise to make clearer
Malawi	Q10	Poor discrimination—CHWs struggled to know that patients should be prioritised over training	Discuss whether the “alternative” should be clearly of lower priority to attending to a sick child
Ghana	Q10	Poor facility and not required in CHW role	Make easier—use a calculation CHWs are required to do regularly on-the-job

Finally, five further structured stakeholder interviews were conducted to determine the validity, fairness and acceptability of the new selection processes (interview schedule in Additional file 2). Participants included CHW supervisors and trainers, human resource managers, ward counsellors and health surveillance assistants who work with CHWs. Interviews were transcribed for analysis and coded by the three themes. All interviews were conducted by the field worker and analysis was undertaken by CB.

Participants reported that the combined content of the test and interview was a good reflection of CHWs’ work, suggesting that the processes have good *content validity*). However, participants in both settings were concerned that those who were not used to written tests or who were particularly nervous may be disadvantaged, creating *potential unfairness*. An additional concern in Malawi was that potential CHWs who could not read and write would not be able to complete the test, but the programme team have included literacy as a key skill for CHWs in their CHW Household Model (see Table 1), because of the need to complete documentation. A small number of stakeholders in Malawi also reported that the use of a formal selection process may help to reduce the bias towards recruitment of CHWs related to Village Chiefs, so could *enhance fairness*. All but one of the participants thought that the new selection process was *acceptable for use* within their CHW programmes. The one participant who disagreed could have had a misconception regarding the programme, because they thought that a selection process was not appropriate with volunteer CHWs (when they are paid).

## Discussion and conclusions

We have described how we developed a selection process for CHWs in two settings in sub-Saharan Africa. We used an intensive process, beginning with the identification of which KSAs to include and going through to testing the selection process with CHWs. The results of beta testing suggested the selection processes are not yet ready for roll-out; in particular, some written test questions require review. A larger-scale evaluation following these refinements would be recommended, including an assessment of predictive validity. However, the lessons learned from going through this process are already being shared with other PIH teams running CHW programmes in other LMICs.

We were fortunate to obtain funding to enable a rigorous development process to be undertaken and appreciate other CHW programmes may not have the resources to do so, even before they are required to find the resources to fund the selection process itself. However, CHW programmes are likely to benefit from investment in selection through improved retention and thus lower re-recruitment and training costs. CHW retention would therefore be important to include in a longitudinal study of predictive validity, particularly given the high attrition rates reported in the literature [13, 14].

Our project drew on good practice from the human resource literature [26] to determine the methods used to develop the selection processes, but there is no gold standard method for doing so; hence, our work had several limitations. Our use of the *Living Goods* selection process in stage 1 was opportunistic and may not have been the best fit for the other two CHW programmes, even had its predictive validity been better. The approaches to data collection used locally differed between settings, so the results of the KSA analysis, for example, are not directly comparable. For pragmatic reasons, the identification of “high-performing” CHWs to include in the observational element was subjective rather than based on explicit performance criteria, and therefore, those identified may not, in reality, be the most highly performing CHWs. No KSAs were identified as being both “essential” and “should be demonstrated at entry to training” by more than half of the stakeholders interviewed (the top right quadrant of Fig. 1b), so we did not learn much from this exercise. We are unsure why this was the case; potentially, the stakeholders interviewed did not have a sufficient understanding of the task or of CHWs’ roles. The number of CHWs included in the alpha and beta tests were small, and for pragmatic reasons, the participants were actual rather than potential CHWs. This was not ideal, as we discovered through the cognitive interviews that participants were using their on-the-job experience to help them answer the questions. The testing itself considered test administration issues in particular, and while we considered whether the questions used were of an appropriate difficulty given the education level of potential applicants, we did not evaluate this robustly.

In the short term, we would recommend the lower intensity approach to selection process development shown in Table 5—although such recommendations need to be considered as being based on our experiences during the study and group reflections on the results, rather than being evidence-based.

**Table 5:** Recommendations for a low-intensity approach to selection process development

The following activities should be included if possible:	
A brainstorming exercise with multiple stakeholders (including CHWs) to identify which KSAs to include in the blueprint and determine an operational selection process design (which includes community involvement) that is both acceptable and feasible;	
Adhering to the recommendations on selection in recent guidelines [22, 23], for example not using age or marital status as selection criteria;	
A search for existing questions/material to include followed by review to ensure suitability in the local context and development of material to fill any gaps in the blueprint;	
Qualitative testing with a small group of CHWs (*N* = 5–10) to check the interpretation of question wording is as intended, plus separate piloting with a further group of CHWs (*N* = 10–20) to quantitatively check that questions are of an appropriate difficulty prior to roll-out (i.e. that not all CHWs get the question incorrect);	
Training of interviewers; and	
A plan for early evaluation using the criteria of validity, reliability, fairness, acceptability and feasibility.	

In the long term, CHW programmes may benefit from continuing to share expertise and research evidence on *what works* in selection [24]. We recommend the establishment of an international database of information about CHW programmes (with similar details to those in the case-studies presented by Bhutta and colleagues [40]) and questions used during selection processes for others to draw on.

Selecting the most appropriate applicants for any role is clearly an important task, for the quality of the human resources available is a key determinant of the quality of any service provided [9]. However, while selection is the foundation of the human resources available for a CHW programme, it has received relatively little attention to date [11, 19, 24]. We have shown that with programme and stakeholder commitment, it is possible to develop selection processes for CHWs that have the potential to improve programme performance. It is now necessary to evaluate whether the processes developed during this project can actually do this.

## Supplementary information

**Additional file 1: **Selection processes used in beta testing

**Additional file 2:.** Data collection templates

## Data Availability

All materials used in data collection have been provided as Additional file 2. The questions used in beta testing and blueprints are shown in Additional file 1. The data collected during alpha and beta testing are pilot data and are not publicly available since they were only used to inform selection process development, but can be obtained from the corresponding author on request.

## Abbreviations

CHW: Community health worker
KSAs: Knowledge, skills and attributes
LMIC: Low- and middle-income country
PIH: Partners In Health
