# Supplementary material for: Case study of a method of development of a selection process for community health workers in sub-Saharan Africa
Source: Hum Resour Health. 2019 Oct 25;17:75. doi: 10.1186/s12960-019-0412-2 (PMC6815009; doi:10.1186/s12960-019-0412-2)
Supplement: Supplementary file 1 — Additional file 1: Selection processes used in beta testing [file 12960_2019_412_MOESM1_ESM.docx]

Appendix 1

Selection processes used in beta testing

**MALAWI: Written test**

**Section 1: Health Numeracy (4 marks)**

1. You are visiting a patient who is supposed to take 1 pill in the morning and 1 pill in the evening. How many pills does the patient need for 28 days? (1 mark) ________________________________
2. Another patient was given 42 pills. This patient needs to take 3 pills per day. After how many weeks should the bottle be empty? ________________________________ (1 mark)
3. You are at the home of a patient. 6 months ago, the patient weighed 65 kilograms. Today, the patient weighs 48 kilograms. How many kilograms has the patient lost? (1 mark; units not necessary) ________________________________
4. You help this patient weighing 48 kilograms get access to medical treatment. At the next home visit, the patient has gained 9 kilograms. How many kilograms does the patient weigh at that home visit? (1 mark; units not necessary)

________________________________

**Section 2: Reading Comprehension and Commitment (4 marks)**

***Please read the story below and answer the 4 questions that follow.***

As a Community Health Worker (CHW), work begins early for Rose. Rose wakes up at 5am. She first goes to the field to farm and feed her livestock. Next, she goes to the borehole to get water for the day, gives her children some likuni phala and makes sure they go to school.

At 10am, she prepares herself to visit her assigned households. On this day, she has planned to visit 4 households. She carries her teaching aids and off she goes. She visits the first house where she screens family members for tuberculosis and children under five years for malnutrition. She goes to the second household where she learns that the woman gave birth at home last night. The baby seems fine, but the mother is bleeding. She still has 2 more households that she needs to visit that day. The following day is the last day to visit all of her households for the month and she had already planned to visit 4 households.

1. Based on the story above, which of the following things did Rose do over the course of the day? (Select one) (1 mark)
2. Help a woman give birth
3. Ride a bicycle
4. Screen for tuberculosis
5. Screen under-5 children for malaria
6. What did Rose take with her as she left for the home visits? (Select one) (1 mark)
   1. Her children
   2. Likuni phala
   3. Teaching aids
   4. Water
7. At the second household, what should Rose have done? (Select one) (1 mark)
8. Proceeded directly to the next 2 households because the baby is well
9. Referred the woman to the hospital and proceed to the 2 remaining households
10. Asked the woman’s mother to look after her and proceed to the 2 remaining households
11. Immediately accompanied the woman and the baby to the hospital
12. Assuming Rose **was unable** to visit the 2 remaining households on that day, what should she **NOT** do on the following day? (Select one) (1 mark)
13. Visit the 4 households she planned for that day
14. Take the day off
15. Visit the 2 households she had missed and 2 of the other 4
16. Visit all 6 remaining households

**Section 3: About You (4 marks)**

***In this section, please imagine you are a CHW***

1. You find 200 Kwacha at the entrance to a household you are visiting. There are no other households nearby. What do you do? (Select one) (1 mark)
   1. Give the money to the household
   2. Give the money to someone who needs it
   3. Give the money to your supervisor
   4. Keep the money
2. A mother from one of your assigned households has sent you a message that her child is sick. You had planned to attend a CHW training session that day. What do you do? (Select one) (1 mark)
3. Pretend you never received the message and attend the training
4. Send a message to the mother that you have a training to attend
5. Visit the household and assess the sick child instead of attending the training
6. Send a message to the mother that you will visit when you are back from the training
7. You have a client that is HIV-positive. You realize that your client recently started dating your neighbor’s daughter and they are talking about getting married. What do you do? (Select one) (1 mark)
8. Tell your neighbor’s daughter to break up with the boyfriend
9. Demand that your client discloses his status to your neighbor’s daughter
10. Encourage your client to disclose his status to your neighbor’s daughter and support him in this process
11. Tell your neighbor’s daughter in confidence that her boyfriend is living with HIV
12. You have a patient who has been diagnosed with TB. This patient does not want to start treatment. You have visited the patient several times to convince him/her but the patient is still refusing. What do you do? (Select one) (1 mark)
    1. Accept their decision not to start treatment
    2. Report them to their pastor
    3. Call your supervisor for advice
    4. Take them to a traditional healer

**MALAWI: Interview**

*DO NOT ASK ANY PROBING QUESTIONS*

*MENTIONING AN ANSWER IS SUFFICIENT TO GAIN CREDIT – DOES NOT REQUIRE ELABORATION*

*Interview time: around 10 minutes; 5 questions*

1. Tell me/us about yourself and what you do. (Not scored)
2. Why do you want to be a Community Health Worker? (2 marks)

| **KSAs = commitment, communication skills (including listening), honesty** | | |
| --- | --- | --- |
| **Excellent = 2** | **Average = 1** | **Bad = 0** |
| Cites 2 or more of the following reasons: interest in health, enthusiasm for learning, contribution to community, working with others, earn a living (must also cite another reason), previous experience working in the community or as a volunteer, other reasons | Cites 1 reason listed under “Excellent” or one other reason (but not only earning a living?) | No insight into role of CHW  Just to earn a living  My community selected me |

1. As a CHW, one of your roles will be to visit households and screen all women of childbearing age for pregnancy and accompany those with a suspected pregnancy to the facility. During one of your visits you find out that a 16-year-old schoolgirl has missed her period. She does not want to come with you to the facility. How would you convince the girl to go to the facility for a pregnancy test and a possible first antenatal care (ANC) visit? (2 marks)

| **KSAs = Communication skills (including listening), persuasion skills, empathy, confidentiality, decision-making** | | |
| --- | --- | --- |
| **Excellent = 2** | **Average = 1** | **Bad = 0** |
| Cites 2 of the following: Displays need to understand what the girl wants and why (understanding context), will talk to girl first, demonstrates or mentions need for empathy, explains the benefits of going to the facility to the patient, offers to support girl through the process regardless of girl’s decision, asks for help from other CHWs/senior | Cites 1 of the answers listed under “Excellent”  Does not break confidentiality | Cites any of the following: Jumps into instruction without understanding girl’s opinion, threatens to (or does) tell parents/family members/school without consent, employs scare tactics |

1. You are at a household visit. The patient asks you a question that you do not know the answer to. What do you do? (2 marks)

| **KSAs = Communication skills (including listening), honesty, working as part of a team, reflectful/seeks help if needed** | | |
| --- | --- | --- |
| **Excellent = 2** | **Average = 1** | **Bad = 0** |
| Says that they do not know the answer **and** will reach out to supervisor/others to find out the answer and get back to the patient | Says they do not know the answer, but do not offer any solution  Only says what they do know about the issue | Makes up an answer; pretend they didn’t hear the question; does not know what to do  Would say what they have heard others say (without knowing whether information is correct) |

1. You saw this in your written test. You have a patient who is HIV-positive. You realize that your patient recently started dating your neighbor’s daughter and they are talking about getting married. Tell me/us about your answer explaining what you would do and your thinking behind it? (4 marks. This question is double-weighted.)

| **KSAs = Communication skills (including listening), honesty, confidentiality, decision-making** | | |
| --- | --- | --- |
| **Excellent = 4** | **Average = 2** | **Bad = 0** |
| Cites 3 of the following: Recognizes complexity of situation, displays empathy and understanding of patient, offers support to patient for disclosing, explores why patient might not want to disclose, understands need for confidentiality, suggests both go for testing together | Cites 2 of the reasons listed under “Excellent,” does not break confidentiality but is very tempted to do so | Does any of the following: Breaks confidentiality, forces person to disclose, threatens to disclose, fails to recognize complexity of situation |

This marks the end of our interview, do you have any questions for me/us?

**GHANA: Written test**

Name: ……………………………………………………………..

Community where you live: ………………………………………

Electoral area (if known): ……………………..…………………..

Age: ……………………………………………………………….

Last school attended: …………..………………………………….

Contact number: …………………………………………………..

**Read the passage below and answer the questions that follow**

Malaria is an acute disease which typically presents with fever, vomiting, headache, joint pains and yellowish eyes. It is a disease of major public health importance and occurs all year round. Some important facts about Malaria are:

1. A child may suffer from Malaria up to five times a year
2. Nearly half of all out-patient attendances in Ghana are due to Malaria
3. One-quarter of all deaths among children under 5 years in Ghana are due to Malaria
4. About 1 in 10 of all pregnant women with Malaria in Ghana are admitted for treatment

Malaria is caused by the Plasmodium parasite, which is transmitted by the female Anopheles mosquito. It is therefore important to prevent mosquito bites. Sleeping under an Insecticide Treated Bednet (ITN) is one of the best ways of preventing mosquito bites; ITNs are therefore a major control strategy in Ghana.

1. What is the *cause* of Malaria? (1 mark)
2. Female mosquitos
3. Plasmodium parasite
4. Anopheles mosquitos
5. When does Malaria occur? (1 mark)
6. Wet season only
7. Dry season only
8. All year round
9. How many times per year would a child *usually* suffer from Malaria? (1 mark)
10. 3
11. 5
12. 10
13. What percentage of deaths in children under 5 years in Ghana are accounted for by Malaria? (1 mark)
14. 15%
15. 25%
16. 50%
17. What major strategy has been adopted in Ghana to prevent Malaria? (1 mark)
18. Hanging an ITN by the window
19. Sleeping under a bednet
20. Sleeping under an ITN

**In the following scenarios imagine you are a CHW**

1. A mother sends you an SMS asking you to visit her child who is sick. You had planned to submit your monthly reports to the district office on the same day which is the deadline for reports. What would you do? (1 mark)
2. Attend to the sick child after submitting your reports
3. Tell the mother you have another sick child to attend to and submit your reports
4. Visit mother and assess the child, then submit your reports
5. You have closed from work and on your way home, when you come across a member of your community who has collapsed by the roadside and there are a lot of people around. You and the people around don’t know how to help him. What would you do? (1 mark)
6. Join the crowd of people who are watching what is happening
7. Call the Community Health Nurse for help
8. Run and tell his relatives
9. After assessing a sick child what would you do? (1 mark)
10. Wipe your hands with a napkin
11. Wear gloves for the rest of the day
12. Wash your hands with soap and running water
13. A mechanic in his dirty working gear comes to your home complaining of severe stomach pain. The pain is so severe that he is unable to stand on his feet. What would you do? (1 mark)
14. Help him to sit down then assess his condition
15. Ask him to remove his overalls before assessing his condition
16. Call his relatives to bring some clean clothes before assessing his condition
17. Your supervisor gives you 50 insecticide treated nets (ITNs) to distribute to children under 5 years old. You distributed 90% of the ITNs. How many ITNs do you have *left*? (Show your working - 2 marks)

……………………………………………………………………………………………………….

1. During your usual home visits, you come across a pregnant woman who plans to deliver her baby at home. There are risks associated with home births. Give one reason *why* it would it be safer for her to deliver at the hospital. (1 mark)

……………………………………………………………………………………………………….

Legibility of handwriting on written test (to be assessed by marker):

1: Legible handwriting

0: Illegible handwriting

**GHANA: Interview**

|  | KSA | Question (Poor-0; Good-1; Excellent-2)  DO NOT PROBE! | 0 | 1 | 2 |
| --- | --- | --- | --- | --- | --- |
| 1 | Appearance | 2: Formal/Smart Casual (skirt at/below the knee, shirt with tie/blouse covering shoulders, full shoes/toes covered, clean hair, clean & short nails)  1: Casual (shirt but no tie, sandals; balanced combination of 2/1/0 indicators)  0: Shabby (t-shirt/polo shirt, flip-flops, dirty hair or nails, untied long hair, long earrings, political statement on clothing/political bands, shirt untucked)  Care needed on scoring to prevent bias |  |  |  |
| 2 | Communication skills | General assessment of communication skills during interview  2: Listens to questions, fluent answers, concise answers, eye contact  1: Some fluency, some eye contact, very quiet (difficult to hear)  0: Interrupts, not fluent answers (jumps from one thing to another), rambling answers, no eye contact |  |  |  |
| 3 | About you | Tell us briefly about yourself.  2: Concise introduction with relevant information (name, education, any relevant experience)  1: Some relevant information, 1-2 pieces of irrelevant information (family details, hobbies etc.)  0: Little or no relevant information, >2 pieces of irrelevant information |  |  |  |
| 4 | Motivation | Why do you want to be a CHW?  2: At least two of: Serve/give back to my community, help people, educate people, career progression/nursing ambition, passion for patients/health care, I think I would be good at it because… (must explain why)  1: One of the reasons above, two reasons above plus one from below, I need to support my family  0: I want the money, I have no job, it’s an easy job |  |  |  |
| 5a | Community Engagement & honesty | Have you participated in any community activities in the last year?  Mention any two  Examples: National sanitation day, community durbar, community volunteering  For each activity: What was your role? When was this held? Who organised the activity?  2: Gives 2 examples that are substantiated  1: Gives 1 example that is substantiated  0: Cannot give any examples  -1: Cannot substantiate one or both examples |  |  |  |
| 6 | Confidentiality | Imagine you are a CHW. An 18 year old unmarried woman confides in you that she is pregnant and that she has not yet told her parents. What would you do?  2: Encourage the girl to go to the health facility for antenatal care AND encourage the girl to tell her parents (or tell them ONLY with her permission).  1: One of the above  0: No idea what to do; would tell the parents without the woman’s permission |  |  |  |
| 7 | Decision-making/ commitment | Three months after being trained as a CHW, you have still not received the bag of equipment you need to undertake home visits. What would you do?  2: Undertake your home visits, improvising where you can and referring where you can’t OR do something else e.g. education, help at the facility AND call your supervisor to let them know about the situation  1: One of the above  0: None of the above; would do nothing; no strategy to solve the problem |  |  |  |
| 8 | Calmness under pressure | This is the last question. Please can you name the Big Six.  Note to interviewer: Observe whether applicant remains calm under pressure – the answer does not matter  2: Maintains composure under pressure, admits does not know  1: Maintains some composure, but clearly panicking a little, fidgets, short laugh  0: Breaks down completely, cries, hysterical laughter |  |  |  |

Selection process blueprints

|  | **Malawi** | | **Ghana** | |
| --- | --- | --- | --- | --- |
|  | Written test | Interview | Written test | Interview |
| Knowledge about health condition/type of care |  |  | X |  |
| Other health knowledge |  |  |  |  |
| Knowledge about the process of obtaining care |  |  |  |  |
| Recognition of need for urgent referral/formal health care |  |  |  |  |
| Accuracy in completion of documentation |  |  |  |  |
| Empathy |  | X |  |  |
| Respect for others/non-discriminatory |  | X | X |  |
| Resilience |  |  |  |  |
| Maintains confidentiality | X | X |  | X |
| Honesty | X | X | X | X |
| Accuracy in using screening tool/taking measurements |  |  |  |  |
| Communication skills (including listening) |  | X |  | X |
| Commitment/motivation - goes above and beyond | X |  | X | X |
| Remains calm under pressure | X |  |  | X |
| Thorough/completes all of a task | X |  |  |  |
| Reflectful/seeks help if needed |  | X |  |  |
| Recognises own health/need to take a break |  |  |  |  |
| Numeracy | X |  | X |  |
| Literacy | X |  | X |  |
| Decision-making | X | X | X | X |
| Working as part of a team | X | X | X |  |
| Persuasion skills (including education) |  | X |  |  |
| Appearance/ personal presentation |  |  |  | X |
| Hygiene/cleanliness (of equipment) |  |  | X |  |
| Use of job/teaching aids |  |  |  |  |
| Confidence |  |  |  |  |
| Time management | X |  | X |  |
