# Supplementary material for: Case study of a method of development of a selection process for community health workers in sub-Saharan Africa
Source: Hum Resour Health. 2019 Oct 25;17:75. doi: 10.1186/s12960-019-0412-2 (PMC6815009; doi:10.1186/s12960-019-0412-2)
Supplement: Supplementary file 2 — Additional file 2:. Data collection templates [file 12960_2019_412_MOESM2_ESM.docx]

Additional file 2: Data collection templates

### CHW OBSERVATION PROFORMA

The proforma has two sections: a daily summary of time use and an activity-specific section, to be completed during each CHW-related activity lasting at least ten minutes (e.g. household visit, accompanyment to a health facility, meeting with supervisor, completing paperwork or checking drug availability). *The use of ten minute intervals will be discussed during training and amended if necessary.* The activities for which the activity-specific section do NOT need be completed are highlighed in the activity code table.

Daily summary of time use (based on Tani, 2016([8](#_ENREF_8)) and programme documentation for Neno and Ghana)

For each ten minute period, please enter the code of the activity being undertaken using the list provided. Use multiple codes if multiple activities occur simultaneously.

A separate activity-specific log shoud be completed for any household visit or other activity expected to last at least 20 minutes, unless the CHW’s client does not wish to have the observer present or if the activity is shaded in the activity code table. Where a client does not wish the observer to be present, please mark the appropriate box with an X. If no information about what happened during an interaction with a client can therefore be obtained, please use code 000X.

CHW ID: Observer ID:

Date:

Location:

**CHW time use**

| **Time** | **Activity code** | **Time** | **Activity code** | **Time** | **Activity code** |
| --- | --- | --- | --- | --- | --- |
| 08.00-08.10 |  | 11.00-11.10 |  | 14.00-14.10 |  |
| 08.10-08.20 |  | 11.10-11.20 |  | 14.10-14.20 |  |
| 08.20-08.30 |  | 11.20-11.30 |  | 14.20-14.30 |  |
| 08.30-08.40 |  | 11.30-11.40 |  | 14.30-14.40 |  |
| 08.40-08.50 |  | 11.40-11.50 |  | 14.40-14.50 |  |
| 08.50-09.00 |  | 11.50-12.00 |  | 14.50-15.00 |  |
| 09.00-09.10 |  | 12.00-12.10 |  | 15.00-15.10 |  |
| 09.10-09.20 |  | 12.10-12.20 |  | 15.10-15.20 |  |
| 09.20-09.30 |  | 12.20-12.30 |  | 15.20-15.30 |  |
| 09.30-09.40 |  | 12.30-12.40 |  | 15.30-15.40 |  |
| 09.40-09.50 |  | 12.40-12.50 |  | 15.40-15.50 |  |
| 09.50-10.00 |  | 12.50-13.00 |  | 15.50-16.00 |  |
| 10.00-10.10 |  | 13.00-13.10 |  | 16.00-16.10 |  |
| 10.10-10.20 |  | 13.10-13.20 |  | 16.10-16.20 |  |
| 10.20-10.30 |  | 13.20-13.30 |  | 16.20-16.30 |  |
| 10.30-10.40 |  | 13.30-13.40 |  | 16.30-16.40 |  |
| 10.40-10.50 |  | 13.40-13.50 |  | 16.40-16.50 |  |
| 10.50-11.00 |  | 13.50-14.00 |  | 16.50-17.00 |  |

**CHW Activity Codes**

*Note that shaded activities do NOT require completion of an activity-specific form.*

General activities

| **Code** | **Activity** |  | **Travel** |
| --- | --- | --- | --- |
|  | **Documentation and Reporting** | 40 | Travel to/from clients’ homes |
| 71 | Documentation (writing in register, client books, monthly reports, referral/accompaniment forms etc.) | 41 | Travel – accompanying client |
|  | **Supervision and training** | 42 | Other CHW-related travel |
| 21 | Visit or phone call from senior, supervisor or programme manager |  | **Village-level activities** |
| 22 | Receiving training | 51 | Planning village meeting |
| 23 | Supervising, monitoring or training others | 52 | Running village meeting |
| 24 | Other supervision and training activity | 53 | Other village-level activity in capacity as CHW |
|  | **Cleaning/organisation** |  | **Non-CHW related activity** |
| 31 | Cleaning, mending, organising or ordering supplies etc. | 61 | Break |
| 32 | Organising workload | 62 | Non-CHW related travel |
| 33 | Other cleaning/organisation activity related to CHW role | 63 | Non-CHW related activity |

CHW role activities

Please code the condition/type of care AND the nature of the task

|  | **Condition/Type of care** |  | **Nature of task** |
| --- | --- | --- | --- |
| 01 | HIV/AIDS | A | Case finding/assessment (including diagnostic tests e.g. RDT for Malaria) |
| 02 | STDs | B | Monitoring (including routine measurements e.g. using MUAC tape) |
| 03 | TB | C | Household counselling/education including health promotion |
| 04 | Family planning | D | Referral |
| 05 | Cholera | E | Accompaniment |
| 06 | NCDs | F | Follow-up |
| 07 | Mental Health | G | Assess domestic conditions |
| 08 | Nutrition | H | Provide resources to promote health (soap, ITN, condoms etc.) |
| 09 | Child health (including immunisations) | I | Provide treatment (ORS/zinc, ACT for Malaria etc.) |
| 10 | Diarrhoea | J | Observe therapy (e.g. DOTS) |
| 11 | Malaria | K | Other |
| 12 | Pneumonia |  |  |
| 13 | Neonatal |  |  |
| 14 | General household visit |  |  |
| 16 | Other |  |  |

Activity-specific knowledge, skills and attributes log (based on CHW programme descriptions for Neno and Ellembelle and multiple sources regarding the competencies of healthcare professionals([37-45](#_ENREF_37)))

CHW ID: Observer ID:

Date: Time:

Location: Activity:

For each CHW-related activity expected to last at least ten minutes please tick if the CHW demonstrated any of the following competencies (tick all that applied for this activity):

| **Competency** | **Demonstrated?** | **Competency** | **Demonstrated?** |
| --- | --- | --- | --- |
| Knowledge about health condition/type of care |  | Empathy |  |
| Other health knowledge |  | Respect for others/non-discriminatory |  |
| Knowledge about the process of obtaining care |  | Resilience |  |
| Recognition of need for urgent referral/formal health care |  | Maintains confidentiality |  |
| Accuracy in completion of documentation |  | Honesty |  |
| Accuracy in using screening tool/taking measurements |  | Reflectful/seeks help if needed |  |
| Communication skills (including listening) |  | Recognises own health/need to take a break |  |
| Commitment/motivation – goes above and beyond |  | Numeracy |  |
| Remains calm under pressure |  | Literacy |  |
| Thorough/completes all of a task |  | Decision-making |  |
| Working as part of a team |  | Persuasion skills (including education) |  |
|  |  | Other (please state): |  |

Is there anything this CHW did that put the client at serious risk of harm? Please provide as much detail as possible:

### Structured interview schedule (Stage 3) - English

*The participant information sheet will include details about the purpose of the interview and its anticipated length and conduct. The interviewer should remind the interviewee of these details, as well as their right to withdraw at any point, prior to commencing the interview. Consent to record the interview should be sought. If the interviewee does not wish the interview to be recorded, then notes should be taken.*

Interview details

Interviewer code: Interviewee code:

Date: Location:

Interview recorded: Yes/No Original language of interview:

Interviewee role

What is your official job title (if relevant)?

How would you describe your current role?

*Probe regarding role in relation to CHW programme if necessary*

The CHW programme and the importance of selecting of the “right” CHWs for programme effectiveness (coding domain: need for selection process)

What do you see as the primary objectives of the CHW programme in Neno/Ellembelle?

Do you think the programme achieves these objectives?

*If not, why not? (Do NOT probe about anything in particular, but follow-up for more detail if the respondent mentions a lack of skills/knowledge/motivation/resilience of CHWs, or other aspects related to WHO is selected as a CHW.)*

What do you know about the current selection process for CHWs in Neno/Ellembelle?

What do you think are the strengths and weaknesses of this approach?

How could it be improved, if at all?

Had different community members been appointed as CHWs, do you think the programme would have the same level of effectiveness as it does currently?

*Why?*

CHW competencies

*We are now going to consider the most important competencies for a CHW in Neno/Ellembelle.*

*Give the list of CHW competencies (from Appendix 3) to the interviewee.*

Here is a list of the knowledge, skills and attributes that may be important for CHWs to possess. In your opinion, are any competencies missing from this list?

*If any are missing, write them on two cards before the next exercises (there will be two decks of cards per interviewee; it will be easiest if these are colour coded; here they are blue and green). You will also have five envelopes, three for exercise 1 and two for exercise 2. One the interviewee has finished sorting, place the cards in the appropriate envelope and ensure that the envelopes are labelled with the interviewees’ code.*

Each card in each of these two decks lists one of the competencies from the list you have just seen.

Please can you sort the blue pile into three categories, representing competencies that it is **essential** for a CHW to have, competencies that it is **important** for a CHW to have and compencies that it would be **nice** if a CHW had.

Please can you sort the green pile into two categories, representing competencies that a CHW **could be trained in** and competencies that a CHW should be able to demonstrate **on entry to training**.

CHW selection tool

*Some CHWs do not perform as well as expected, and others leave their posts shortly after training. It is possible that a modified selection process might help to ensure that only the “best” and committed CHWs take up posts. We seek your views on two potential new methods, which would be used alongside existing community engagement in selection and would not replace it.*

*Show the Living Goods written test to the interviewee and allow them time to read it. Also provide a copy of existing literacy test (Neno).*

How would you feel about a written test such as that used by *Living Goods* being used as part of the selection process for CHWs? (coding domain: acceptability)

Would it be more or less acceptable to you than the existing test? (coding domain: acceptability)

How does the content of the *Living Goods* test compare to that of the current test in terms of the competencies sought in a CHW? (coding domain: validity)

Would the content of the *Living Goods* test be appropriate for Neno/Ellembelle – as is or with modification? (coding domain: validity)

How easy or difficult do you think potential CHWs in Neno/Ellembelle would find this test? (coding domain: validity)

How does the level of difficulty compare with that of the existing test? (coding domain: validity)

Do you think any potential CHWs might be disadvantaged if this type of test was used even though they are likely to be good CHWs? (coding domain: potential for bias)

*If so, who/which group of CHWs and why?*

For each group of CHWs (e.g. males comapred with females), then ask:

Do you think any disadvantage would be different to that for the existing test? (coding domain: potential for bias)

This test is currently designed to be completed using pencil and paper. Would you use the same approach in Neno/Ellembelle? (coding domain: feasibility)

*If no, what approach would you use?*

How long do you think potential CHWs in Neno/Ellembelle should be given to complete this test? (coding domain: feasibility)

What would be the right length for a written test such as this? (*The answer could be in terms of number of questions or in time.)* (coding domain: feasibility)

*Show the Living Goods interview schedule to the interviewee and allow them time to read it.*

How would you feel about a face-to-face interview such as this being used as part of the selection process for CHWs? (coding domain: acceptability)

How might you modify the content so it would be more appropriate for use in Neno/Ellembelle? (coding domain: validity)

How well do you think potential CHWs in Neno/Ellembelle would be able to answer the questions? (coding domain: validity)

Do you think any potential CHWs might be disadvantaged if a face-to-face interview was used even though they are likely to be good CHWs? (coding domain:potential for bias)

*If so, who and why?*

What would be the most appropriate length (in minutes) for a face-to-face interview? (coding domain: feasibility)

Who should be included on the interview panel (e.g. CHW, senior CHW, community leader, community member, programme manager)? (coding domain: feasibility)

*To CHW programme providers only*:

Do you know how much you currently spend on selection of CHWs? If so, how much and what does this include and for how many CHWs (confirm if per applicant or per CHW recruited)?

Once a selection process has been developed, what is the maximum amount you would be willing to spend on CHW selection, per CHW recruited? Does this include the cost of indivdiuals’ time e.g. programme managers, senior CHWs? (coding domain: feasibility)

Other information and close of interview

Is there anything else you would like to add? (code as appropriate)

*Thank the interviewee for their time; offer token of appreciation where appropriate.*

### Structured interview schedule (Stage 5) - English

*The participant information sheet will include details about the purpose of the interview and its anticipated length and conduct. The interviewer should remind the interviewee of these details, as well as their right to withdraw at any point, prior to commencing the interview. Consent to record the interview should be sought. If the interviewee does not wish the interview to be recorded, then notes should be taken.*

Interview details

Interviewer code: Interviewee code:

Date: Location:

Interview recorded: Yes/No Original language of interview:

Interviewee role

How would you describe your current role? What is your official job title (if relevant)?

*Probe regarding role in relation to CHW programme if necessary*

The CHW programme and the importance of selecting of the “right” CHWs for programme effectiveness (coding domain: need for selection process)

What do you see as the primary objectives of the CHW programme in Neno/Ellembelle?

Do you think the programme achieves these objectives?

*If not, why not? (Do NOT probe about anything in particular, but follow-up for more detail if the respondent mentions a lack of skills/knowledge/motivation/resilience of CHWs, or other aspects related to WHO is selected as a CHW.)*

Had different community members been appointed as CHWs, do you think the programme would have the same level of effectiveness as it does currently?

*Why?*

CHW selection tool

*Some CHWs do not perform as well as expected, and others leave their posts shortly after training. It is possible that a modified selection process might help to ensure that only the “best” and committed CHWs take up posts. We seek your views on two potential new methods, which would be used alongside existing community engagement in selection and would not replace it.*

*Show the beta version of the written test to the interviewee and allow them time to read it.*

How would you feel about a written test such as this being used as part of the selection process for CHWs? (coding domain: acceptability)

How might you modify the content so it would be more appropriate for use in Neno/Ellembelle? (coding domain: validity)

How easy or difficult do you think potential CHWs in Neno/Ellembelle would find this test? (coding domain: validity)

Do you think any potential CHWs might be disadvantaged if this type of test was used even though they are likely to be good CHWs? (coding domain: potential for bias)

*If so, who and why?*

How long do you think potential CHWs in Neno/Ellembelle should be given to complete this test? (coding domain: feasibility)

*Show the beta version of the interview schedule to the interviewee and allow them time to read it.*

How would you feel about a face-to-face interview such as this being used as part of the selection process for CHWs? (coding domain: acceptability)

How might you modify the content so it would be more appropriate for use in Neno/Ellembelle? (coding domain: validity)

How well do you think potential CHWs in Neno/Ellembelle would be able to answer the questions? (coding domain: validity)

Do you think any potential CHWs might be disadvantaged if a face-to-face interview was used even though they are likely to be good CHWs? (coding domain: potential for bias)

*If so, who and why?*

Other information and close of interview

Is there anything else you would like to add? (code as appropriate)

*Thank the interviewee for their time; offer token of appreciation where appropriate.*

### Cognitive interviews - English

*The participant information sheet will include details about the purpose of the interview and its anticipated length and conduct. The interviewer should remind the interviewee of these details, as well as their right to withdraw at any point, prior to commencing the interview. Consent to record the interview should be sought. If the interviewee does not wish the interview to be recorded, then notes should be taken.*

Instructions to CHWs: We would like you to complete this test/tool. As you answer each question, please can you tell me why you are giving a particular answer. Before you move onto the next question, I will ask you some follow-up questions. Please take as long as you need for each question; there is no time limit.

After each question:

(If needed): Why did you give that answer?/How did you arrive at that answer? (Version depending on what is being asked).

Was this question easy or hard to answer?

Have you ever had to answer a similar question/do a similar task in your role as a CHW?

(If “yes”): Please can you tell me more about when you had to do so?

(If “no”): Do you think this question is relevant for CHWs?

Is there anything else you would like to add? (code as appropriate)

*Thank the interviewee for their time; offer token of appreciation.*
